# Massive Habitat-Specific Genomic Response in *D. melanogaster* Populations during Experimental Evolution in Hot and Cold Environments

**DOI:** 10.1093/molbev/mst205

**Published:** 2013-10-22

**Authors:** Ray Tobler, Susanne U. Franssen, Robert Kofler, Pablo Orozco-terWengel, Viola Nolte, Joachim Hermisson, Christian Schlötterer

**Affiliations:** ^1^Institut für Populationsgenetik, Vetmeduni Vienna, Vienna, Austria; ^2^Mathematics and Biosciences Group, Department of Mathematics, University of Vienna, Vienna, Austria; ^3^Max F. Perutz Laboratories, Vienna, Austria

**Keywords:** experimental evolution, *D. melanogaster*, adaptation, standing genetic variation, next-generation sequencing, evolutionary genomics

## Abstract

Experimental evolution in combination with whole-genome sequencing (evolve and resequence [E&R]) is a promising approach to define the genotype–phenotype map and to understand adaptation in evolving populations. Many previous studies have identified a large number of putative selected sites (i.e., candidate loci), but it remains unclear to what extent these loci are genuine targets of selection or experimental noise. To address this question, we exposed the same founder population to two different selection regimes—a hot environment and a cold environment—and quantified the genomic response in each. We detected large numbers of putative selected loci in both environments, albeit with little overlap between the two sets of candidates, indicating that most resulted from habitat-specific selection. By quantifying changes across multiple independent biological replicates, we demonstrate that most of the candidate SNPs were false positives that were linked to selected sites over distances much larger than the typical linkage disequilibrium range of *Drosophila melanogaster*. We show that many of these mid- to long-range associations were attributable to large segregating inversions and confirm by computer simulations that such patterns could be readily replicated when strong selection acts on rare haplotypes. In light of our findings, we outline recommendations to improve the performance of future *Drosophila* E&R studies which include using species with negligible inversion loads, such as *D. mauritiana* and *D. simulans*, instead of *D. melanogaster*.



## Introduction

Understanding the process of adaptation at the genomic level remains one of the fundamental questions for 21st century biology (Stapley et al. 2010). The arrival of next-generation sequencing (NGS) technology has ushered in a new era of adaptation research: by allowing essentially complete genome sequence information to be assayed at the population level, the prospect of identifying the majority of causal loci underlying adaptation is now foreseeable. To realize this objective, a series of recent studies have leveraged the power of NGS in conjunction with experimental evolution (Bergelson and Roux 2010; Parts et al. 2011; Dettman et al. 2012; Orozco-terWengel et al. 2012). The experimental evolution framework allows the study of phenotypic and genetic changes in experimental populations under environmental and demographic parameters of the experimenters’ choice (Kawecki et al. 2012). Thus, the combination of NGS and experimental evolution—a system dubbed evolve and resequence (E&R; Turner et al. 2011)—provides a flexible and powerful approach to elucidate population genomic responses to adaptation.

Many recent E&R studies have utilized *Drosophila melanogaster* to elucidate the genomic architecture of specific traits (Burke et al. 2010; Turner et al. 2011; Zhou et al. 2011; Orozco-terWengel et al. 2012; Remolina et al. 2012; Turner and Miller 2012; Turner et al. 2013). In addition to being a model organism for genetic research, *D. melanogaster* also has the advantage of having a well-annotated reference genome and a large collection of mutant and RNAi lines. Furthermore, natural populations of *D. melanogaster* maintain abundant polymorphism with relatively low levels of linkage disequilibrium (LD) (Mackay et al. 2012), which is expected to result in high-resolution genomic maps. A common feature of published *D. melanogaster* E&R studies is that a very large number of SNPs (at least hundreds) are identified as targets of selection (i.e., candidate SNPs). Theoretical arguments suggest that, when taking into account the large frequency changes that are reported for candidate SNPs, it is unlikely that all of these sites could be independent targets of selection (Haldane 1957; Nunney 2003). Rather, it appears that current methods are confounded by large numbers of false positives. The reasons why, however, remain elusive.

To shed light on why such large numbers of candidate SNPs are typically found in *D. melanogaster* E&R studies, we subjected multiple replicates of the same starting population to two different selection regimes, a hot and a cold environment. In accordance with previous studies, we detected thousands of putative selected sites for both environments, although there was relatively little overlap between the two sets of candidates. By leveraging information from additional independent replicates and the starting allele frequencies of candidate SNPs, we demonstrate that our populations carry signatures of environmentally dependent thermal selection. Moreover, we demonstrate that most of the candidate SNPs were false positives that were linked to selected sites over distances much larger than those typically observed for *D. melanogaster*, with many being coincident with large inversions that underwent sizeable frequency shifts during the experiment. Such long-range associations were readily reproduced in simulations that replicated common features of published *Drosophila* E&R studies, indicating that this phenomenon may be a general cause of false positives in these studies. Based on our results, we make several suggestions intended to improve E&R performance, which include substituting *D. melanogaster* for sibling species with few reported inversions such as *D. simulans* or *D. mauritiana*.

## Results

Ten replicated *D. melanogaster* populations were established from 113 recently collected isofemale lines and maintained at a census size of 1,000 individuals with nonoverlapping generations. These were evenly split between two different temperature regimes—the “hot” and “cold” treatments—that cycled between 18–28 °C and 10–20 °C, respectively. Genomic DNA was sequenced from pools of ∼500 adult females (Pool-Seq) for three different replicates in each evolved treatment (∼generation 15) and the starting population (i.e., the “base” population). After quality filtering, ∼1.45 million SNPs were identified in our populations, a number that is consistent with previous reports for *D. melanogaster* (see Materials and Methods for more details on the experimental regime, sequencing protocols, and SNP calling).

### Candidate SNPs

We identified putative selected sites in each treatment—that is, candidate SNPs—by comparing the *P* values from the Cochran–Mantel–Haenszel (CMH) test (Agresti 2002) for each observed SNP against those for sites simulated under forward Wright–Fisher evolution (see Materials and Methods). Using an empirical false discovery rate (FDR) of 0.001 and a simulated *N_e_* of 250, we identified ∼17,000 candidate SNPs in the hot treatment and ∼3,500 candidate SNPs in the cold treatment (hereafter referred to as hot candidate SNPs and cold candidate SNPs, respectively; supplementary table S1, Supplementary Material online). Because our simulations do not account for linkage between sites and therefore may result in biased estimates, we performed additional simulations of neutral evolution among segregating haplotypes. By allowing for recombination between the different haplotypes, these simulations explicitly model correlated allele frequency dynamics caused by linkage between sites (see Materials and Methods). The haplotype-based simulations did not reduce the number of inferred candidates nor did removal of regions with low recombination rates (<2 cM/Mb) produce notable differences (supplementary table S1, Supplementary Material online). This suggests that our assumption of independence among SNPs did not upwardly bias the inferred number of candidates. Thus, despite using a conservative significance threshold and being robust to false positives caused by drift in low recombining regions, the detection of such a large number of candidate SNPs suggests that our estimates for both treatments were probably inflated by a large number of false positives.

### Different Selection Signatures in the Two Thermal Regimes

**Fig. 1.**
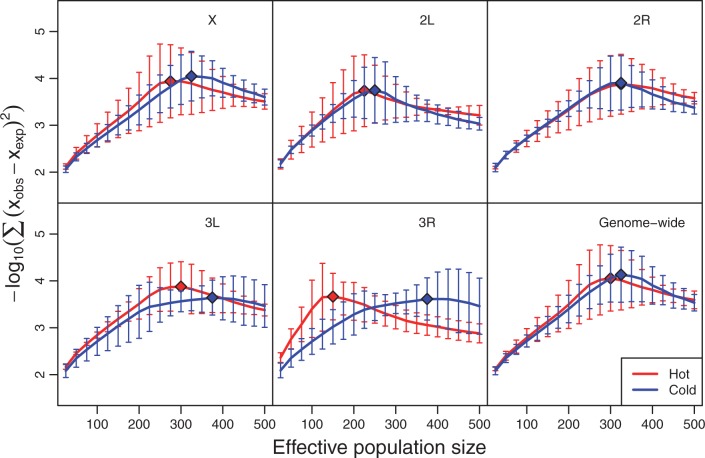
Estimates of effective population size in the evolving populations. The sum of the mean squared differences (negative log_10_ transformed) between the allele frequency change distributions for the experimental and simulated data for a range of *N_e_* values. Larger values on the *y* axis indicate a better fit between the observed and simulated values and the diamond indicates the single best fit overall; that is, the best estimate of *N_e_* for a given chromosome arm or whole genome. Error bars are the standard errors for the three replicates.

Reasoning that differences in the efficacy of selection would be discernable through differences in effective population size (*N_e_*), we estimated the *N_e_* for each chromosome arm in both treatments (see Material and Methods). Indeed, *N_e_* was consistently larger for the cold treatment relative to the hot treatment (fig. 1). This suggests that the effects of selection—either acting directly on each SNP or via linkage between selected and neutral sites—was more pervasive in the hot treatment overall. Moreover, the estimated *N_e_* of the X-chromosome was higher than that for most autosomes in both treatments. Because the *N_e_* of the X-chromosome is expected to be 25% smaller than that of the autosomes in neutrally evolving populations, our results suggest that selection was more influential on the autosomes in both treatments. Although we cannot rule out the possibility that the intertreatment difference in *N_e_* was caused by environmentally induced nongenetic fitness variation, it is unlikely that this mechanism was responsible for the *N_e_* variation that was observed across chromosome arms within each treatment. Thus, our results suggest that selection affected a large number of SNPs during the experiment, potentially in a habitat-specific manner.

**Fig. 2.**
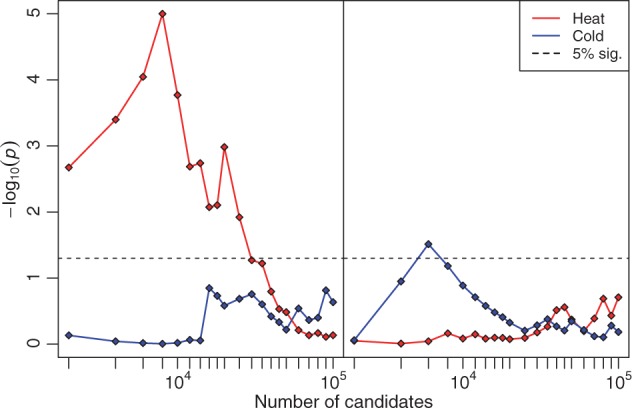
Enrichment of candidate SNPs within genes associated with heat and cold tolerance in *Drosophila*. Successively larger sets of candidate SNPs from the hot (left panel) or cold (right panel) treatment were tested for enrichment in gene sets either associated with cold or heat tolerance. All points above the dashed line indicate that the probability of obtaining the same number of thermal tolerance genes with at least one candidate SNP by chance was less than 5%. All genes were taken from the CESAR database and categorized according to whether they were uniquely associated with either heat or cold tolerance. The enrichment tests indicate that selection operated in the expected direction, although the large number of candidate SNPs for which a significant enrichment was found implies that these were confounded by false positives.

To evaluate the hypothesis that different genes were targeted in the two selection regimes, we tested whether outlier SNPs were enriched among a carefully curated data set of genes associated to either heat or cold stress (see Materials and Methods). To avoid biasing results by making an a priori assumption about the best number of candidate SNPs in each treatment, enrichment was tested over successively larger sets of candidates, ranging from the top 2,000 to 100,000 “candidate” SNPs. The results show that hot candidate SNPs were highly enriched in heat tolerance genes and that cold candidate SNPs were moderately enriched within cold tolerance genes (fig. 2). Notably, there was an order of magnitude more genes associated with heat tolerance than cold tolerance in our curated data sets (∼320 vs. ∼20; supplementary table S2, Supplementary Material online), such that the latter may have suffered from reduced power caused by having a relatively poorly characterized pathway. In contrast, there was no significant enrichment of hot candidate SNPs in the cold tolerance genes or of cold candidate SNPs in heat tolerance genes. Thus, although these results confirmed treatment-specific selection, we still observed associations for sets of candidates that were even larger than those estimated using our conservative FDR cutoff. This result reaffirms that we have a large number of false positives that need to be accounted for.

**Fig. 3.**
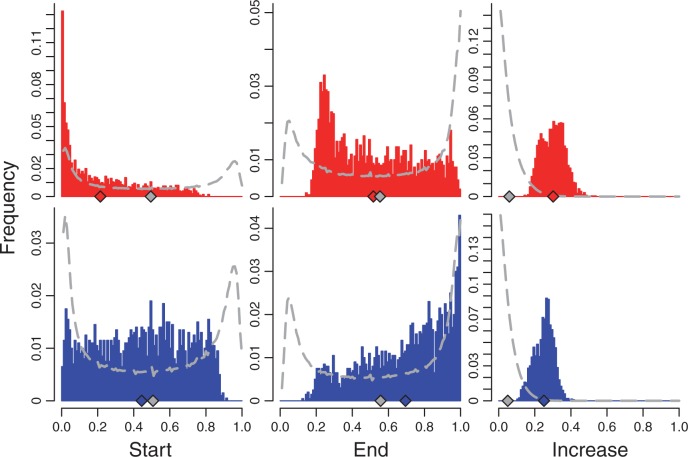
Allele frequency distribution of candidate SNPs. Distribution of the starting (left panels), end (middle panels), and increase (right panels) in allele frequencies for the top 2,000 candidates SNPs in the hot (top row) and cold (bottom row) treatment. Alleles are polarized by the rising allele in the derived treatment, such that plots display the frequencies of the putative selected alleles. The dashed gray line indicates the distribution of all ∼1.45 million SNPs, and the diamonds on the *x* axis indicate the mean frequency for candidate (red/blue) and all (gray) SNPs.

Natural *D. melanogaster* populations have been described to vary seasonally. This pattern was first reported for morphological traits (Tantawy 1964) and was more recently extended to inversions (Stalker 1980) and SNPs (Bergland et al. 2013) that exhibited cyclic frequency changes across seasons. Molecular evidence suggests that alleles that had been selected over the winter remained at relatively high frequencies in *Drosophila* populations collected in the spring before decreasing again by the fall (Bergland et al. 2013). Because the founder population of our experiments was collected in the early summer, it probably more closely resembles a winter/spring population than a fall population (Bergland et al. 2013). Therefore, we reasoned that alleles involved in adaptation to cold or hot environments may start at high or low frequencies, respectively, whereas false positives are expected to follow the background frequency distribution of neutral SNPs. When conditioning the allele frequency on the rising allele—the allele expected to be coming under selection—we observed an excess of low-frequency alleles in the hot treatment, and conversely an enrichment of intermediate frequency alleles in the cold treatment (fig. 3). Given that the high-frequency alleles experience very little allele frequency change before fixation, the lack of predicted high-frequency alleles among the cold candidates is likely to be a direct consequence of insufficient power to detect such SNPs. Notably, this pattern was not restricted to the top 2,000 candidate SNPs but extends to a much larger fraction of loci (supplementary fig. S1, Supplementary Material online). This further reinforces the idea that a large number of SNPs are either directly selected or behave similar to selected loci in each treatment.

### Reproducibility of Ranked SNPs

**Fig. 4.**
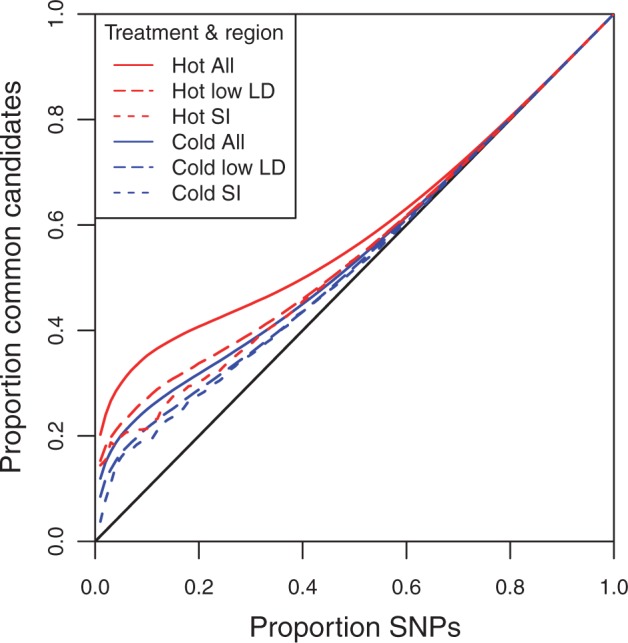
Concordance between two independent groups of replicates. Modified ROC curve showing the proportion of common candidate SNPs shared between two independent sets of derived populations relative to cumulatively larger sets of ranked SNPs (see Materials and Methods) for both hot (red lines) and cold (blue lines) treatments. Separate lines are plotted for full data set (∼1.45 million SNPs; All), as well as for those SNPs only falling in regions of putatively low LD (i.e., regions outside of inversions and low recombining regions) or for short introns in these regions (SI). Lines above the black diagonal line indicate that there are more common candidate SNPs than expected by chance, such that our top candidates are more reproducible than those neutrally evolving populations. Because sites with low LD or in short introns with low LD show a similar reduction in concordance relative to all sites, much of the excess concordance is probably due to changes in inversions and long-range LD between selected and neutral sites.

A testable consequence of directional selection is that targeted alleles are expected to increase in frequency across all replicates, whereas false positives should not replicate. Hence, to further distinguish between true positives and noise among our top candidates, we sequenced two additional F15 replicates in each treatment and quantified the level of reproducibility between these and the original replicates. Reproducibility was measured using a modified receiver operating characteristic (ROC) curve, which visualizes the excess of shared SNPs of similar rank (rank being assigned by *P* value) between our original and new data sets (see Materials and Methods). For both temperature regimes, we noted a substantial concordance between the independent replicates, as indicated by the ROC curve being clearly above the diagonal (fig. 4). In contrast, neutrally evolving sites that were simulated to have parameters similar to our treatment data, and which included recombination, did not differ from expectation (supplementary fig. S2, Supplementary Material online; see Materials and Methods). Nonetheless, because the concordance between the independent sets of replicates peaked among the top 50,000–100,000 ranked SNPs in each treatment, this analysis could not further distinguish truly selected SNPs from false positives.

The high concordance that we observed could have resulted from strong LD between selected and linked neutral SNPs, which is expected to produce correlated allele frequency changes across contiguous sites. We tested this hypothesis by reanalyzing the data after removing low recombining regions and genomic regions known to contain segregating cosmopolitan inversions (see Materials and Methods). The resulting ROC curve exhibited a substantially reduced signal of concordance between the two independent sets of replicates but was still greater than expected by chance (fig. 4). Next, we repeated the analysis using only short intronic sites (without splicing signals) within the putative low LD regions. Evidence suggests that short introns are unlikely to carry selectively favored alleles in *D. melanogaster* (Halligan and Keightley 2006; Parsch et al. 2010; Clemente and Vogl 2012), whereby these regions are good proxies for neutral loci (but see Farlow et al. 2012). Although SNPs in short introns were not expected to show much consistency across the groups, the signal of concordance was similar to that of the putative low LD regions (fig. 4). Consequently, this pattern indicates that much of the selection signal could be generated from linkage between neutral sites and the true targets of selection.

### Effects of Long-Range LD on False Positives

**Fig. 5.**
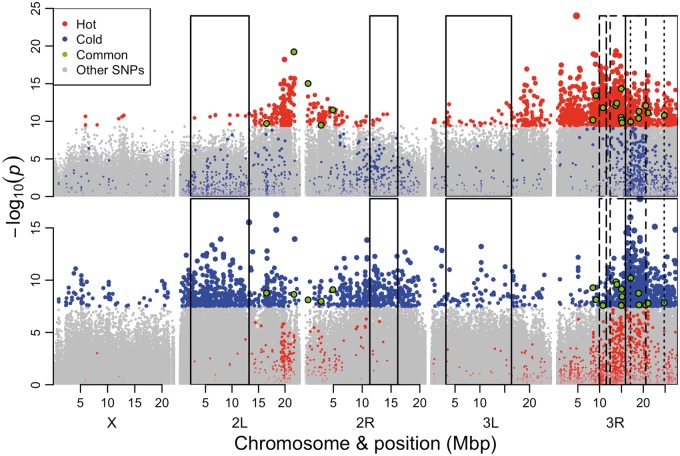
Manhattan plots showing the genomic distribution of candidate SNPs. The negative log_10_-transformed *P* values of all SNPs relative to genomic position. The top 2,000 candidate SNPs for the hot (top panel) and cold (bottom panel) treatment are highlighted (see key). Candidates are categorized as being unique to the hot (red) or cold (blue) treatments or common to both (green). Point size scales with significance, except for common SNPs, which are shown at a standard size for emphasis. Inset boxes indicate the position of inversions and a putative selected haplotype block on 3R (large dashes; most proximal to centromere on 3R). The inversions are as follows: In(2L)t, In(2R)NS, In(3L)P, In(3R)Payne (short dashes), In(3R)C (dots), and In(3R)Mo (solid line). The candidate SNPs typically appear as either isolated points or arranged in broad, flattened humps and tend to be clustered on 3R where three large inversions overlap. These patterns further imply that inversions and long-range LD have generated many false positives in the study.

**Fig. 6.**
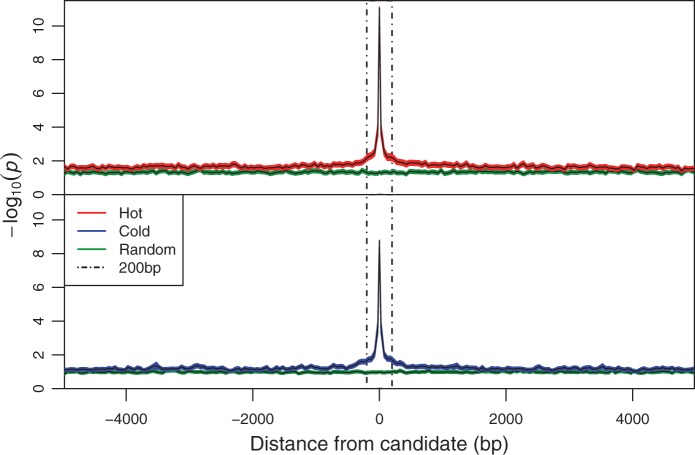
Correlation between the top 2,000 candidate and flanking SNPs. The decay in LD (correlation) around candidates is measured as the rate of decline in neighboring (negative log_10_ transformed) *P* values to background levels. Data are averaged over successive 50-bp bins flanking focal SNPs. Solid black lines indicate the bin means. The red, blue, and green polygons around these lines indicate 95% confidence intervals for hot treatment, cold treatment, and random (i.e., background) SNPs, respectively. In accordance to reported results from *D. melanogaster*, most of the LD is lost within 200 bp either side of each focal SNP (indicated by dashed lines).

In genome-wide association studies (GWAS), linkage between selected and neighboring neutral sites results in a characteristic pattern observed in Manhattan plots, namely a series of adjacent sites with significant *P* values that are arranged into a peak. Such patterns were not readily recognizable in either treatment in our study, however. Rather, the top candidates occurred as either isolated SNPs or broad flattened humps (fig. 5). Furthermore, analyses of genomic regions flanking candidate SNPs showed that LD had a limited range beyond the putative selected site (fig. 6). This pattern is consistent with the low levels of LD observed in *D. melanogaster* populations (Miyashita and Langley 1988; Andolfatto and Wall 2003; Mackay et al. 2012) and has been previously interpreted as evidence against linkage as a possible cause for the large number of candidate SNPs in *Drosophila* E&R studies (Orozco-terWengel et al. 2012). To reconcile the anomaly of thousands of putative selected SNPs being detected despite an apparent lack of LD in our study, we propose that associations beyond 200 bp—which is widely regarded the limit of LD in *D. melanogaster* (Mackay et al. 2012)—may be prevalent within our experimental populations.

Support for this idea comes from two separate findings. First, the excess concordance observed for short introns outlined above is still evident after exclusion of intronic sites located in the same genes that contained one or more candidate SNPs. Thus, although excluding these intronic sites did reduce concordance to some extent, the excess concordance was still visible even after removing genes containing one or more of the top 100,000 candidate SNPs (supplementary fig. S3, Supplementary Material online). Given that short introns are not expected to contain selected sites (Parsch et al. 2010; Clemente and Vogl 2012), we suggest that linkage between sites in short introns and selected sites in regions other than the enclosing gene are responsible for producing this pattern.

Second, a good, albeit potentially extreme, example of long-range LD is provided by a 1 Mb region located on an inversion-free part of chromosome 3R that was first described by Orozco-terWengel et al. (2012). Among our genome-wide top 2,000 hot candidates, 281 were located in this region, which represents a massive enrichment relative to its size. These sites increased in frequency by ∼27% over the first 15 generations on average, and 33% of the 24K SNPs in this region had the same rising allele across all five replicates in the hot treatment (cf. 23% across the rest of the genome, *P* value <2.2e−16 using Fisher’s exact test). Furthermore, a large number of putative selected alleles in this region had starting frequencies <1% (118 SNPs), and these sites displayed highly homogeneous increases in frequency during the study (supplementary fig. S4, Supplementary Material online). Thus, although it is evident from the data that this is a low-frequency haplotype that increased across all replicates in the hot treatment, 83% of the putative candidates in this region were separated by distances greater than 200 bp, with an average distance of 5,570 bp between sites. Because such a broad spacing between candidate sites is well beyond the typical range of LD in *D. melanogaster*, we suggest that long-range LD is responsible for these patterns and the majority of false positives more generally.

**Fig. 7.**
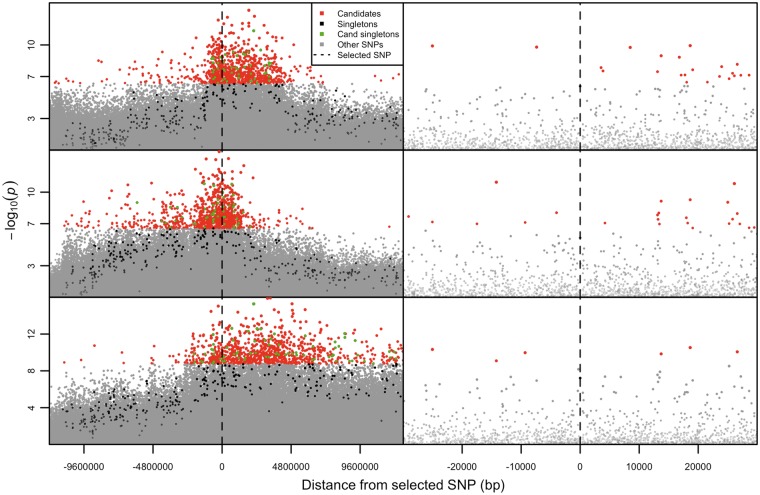
Simulated long-range LD generated by a singleton under strong selection. Manhattan plots showing the distribution of the top 1,000 candidate SNPs from three separate sets of simulated data (top to bottom panels) in which a singleton carried by a single chromosome-length haplotype is under strong selection (see Materials and Methods). The full chromosome is shown in the panels on the right, with a zoomed-in view shown in the panels on the left. Significant SNPs occur vast distances from the selected site (dashed line) and tend to be several thousands of base pairs apart. Thus, it appears likely that long-range LD between selected and neutral sites can readily inflate the number of false positives in an E&R study.

To evaluate to what extent long-range LD could be observed in a typical E&R study, we simulated strong selection on a single allele private to a rare haplotype using allele frequency changes and parameters similar to those in the hot treatment (see Materials and Methods). We found that neighboring candidate SNPs were often separated by several thousand base pairs or more and could be millions of base pairs from the selected site (fig. 7). These results clearly indicate that long-range LD can be readily generated under a simple but plausible scenario for E&R studies.

## Discussion

Exposure of *D. melanogaster* to novel hot or cold environments resulted in selection of alleles in thermal tolerance genes specific to each environment. Moreover, the starting frequencies of the putative selected SNPs differed markedly between the two environments, suggesting that they had experienced selection within the natural progenitor population prior to the start of the experiment. Hence, our study reveals that a potentially rich source of information lies in using the base population information, which has not been considered in most published E&R studies to date.

Despite strong evidence that differential thermal selection contributed to our top candidates in each treatment, the shear number of putatively selected sites indicates that our results were confounded by a large number of false positives. Given that short-range LD and strong drift in low recombining regions were unlikely to be major contributors to the excess of false positives in our study, we hypothesized that LD over longer distances was responsible for much of this excess. Long-range LD in our experimental populations could have arisen as a byproduct of the founders being a small sample of the much larger natural population. In this case, chance correlations between sites separated by vast distances may result from the finite sampling of alleles. Long-range LD may also originate within natural populations. For instance, strong LD between loci separated by large distances has been reported for Japanese *D. melanogaster* populations (Takano-Shimizu et al. 2004; Itoh et al. 2010). Because the LD was much stronger in the spring than in the autumn, this finding was attributed to exaggerated sampling effects during the previous winter bottleneck, although there was evidence that recurrent epistatic selection was also responsible for some linked loci. Finally, the introgression of new haplotypes from migrant individuals is another natural process that could produce long-range LD. Recent and ongoing admixture has been reported for African (Pool and Aquadro 2006; Pool et al. 2012) and North American (Caracristi and Schlötterer 2003) *D. melanogaster* populations, suggesting that, in principle, this may also contribute to long-range LD in natural populations.

The systematic increase in frequency of alleles in the hot treatment that were broadly distributed across a 1 Mb region of 3R demonstrates the effects of long-range LD in our experiment. In combination with differences in the distribution of the starting allele frequencies, long-range LD may also help to explain the differences in the number of candidates and the level of concordance observed between the two treatments. Given that most candidates were rare in the hot treatment and common in the cold treatment—presumably resulting from prior selection against (for) heat (cold) tolerance alleles—long-range LD is expected to be more extensive among the hot candidates. This is because rare haplotypes are expected to harbor many more private SNPs than common ones, and all such sites will experience the same change in allele frequency until these associations are broken down by recombination. The difference in *N_e_* between the two treatments is also consistent with more extensive long-range LD producing more widespread changes in allele frequencies in the hot treatment.

Segregating inversions are another probable source of false positives in our study. The large decrease in concordance that was observed when inversions and regions with low recombination were removed from the data set supports this idea (fig. 4). Additional confirmation comes from a recent study which found that several large inversions were segregating in our populations over the course of the experiment, including three partially overlapping inversions on chromosome arm 3R (Kapun et al. 2013). By tracing the trajectory of alleles that were private to each inversion, a single inversion was found to increase across all three replicates in each treatment (In(3R)c by 25% in the hot and In(3R)mo by 15% in the cold). Indeed, the majority of the top 2,000 candidate SNPs was observed on chromosome arm 3R for both treatments (∼80% for the hot and ∼40% for the cold; supplementary table S3, Supplementary Material online) with many candidate SNPs lying within these inversions. Furthermore, the greatest concentration of cold candidates was situated within the region where all three inversions overlap. Because recombination is likely to be suppressed in this region, the local effects of LD and drift are expected to be exacerbated relative to the rest of the genome. Thus, changes in inversion frequencies are also likely to have substantially inflated the number of false positives in our study.

### Implications for Future E&R Research

Our results indicate that E&R studies with small founder populations, strong selection, and large segregating inversions are likely to suffer from large numbers of false positives. These factors are likely to be present in other *D. melanogaster* E&R studies—indeed, several large inversions segregate at intermediate frequencies in natural *D. melanogaster* populations (Corbett-Detig and Hartl 2012; Kapun et al. 2013)—meaning that our findings have important implications for future E&R research in *Drosophila*.

First, if seasonal selection is common in *D. melanogaster*, which accumulating evidence suggests may be the case (Stalker 1980; Takano-Shimizu et al. 2004; Itoh et al. 2010; Bergland et al. 2013), then the genetic composition of the experimental populations will be dependent on their time of collection. Evidence suggests that genes associated with stress resistance are highly pleiotropic in *Drosophila* (Rose et al. 1992; Hoffman et al. 2001; Bubliy and Loeschcke 2005; Kolss and Kawecki 2008), implying that a broad spectrum of traits may be affected in this way.

Second, large numbers of false positives are expected in E&R studies that use small founder populations as a direct result of long-range LD between selected and neutral sites. The effect of founder population size on the performance of experimental design has been explicitly addressed in a recent haplotype-based simulation study (Kofler and Schlötterer 2013). The authors demonstrate that both the power and specificity of E&R studies is increased when larger numbers of founders are used, with the gains in performance being particularly noticeable when the strength of selection per SNP is strong. Another plausible way to reduce the impact of long-range LD is to create independent replicates from different sets of founders. Although this does not remove long-range LD per se, it results in different associations across replicates, such that analyses that combine replicates should remove the majority of such effects.

Finally, our results indicate that inversions are likely to be a major hindrance in detecting causal loci. Large segregating inversions appear to be ubiquitous in natural *D. melanogaster* populations, and recent work suggests that newly introduced inversions may influence genetic diversity across entire chromosome arms, presumably by suppressing recombination well beyond inversion breakpoints (Corbett-Detig and Hartl 2012; Pool et al. 2012; Kapun et al. 2013). Consequently, any improvements in performance that might be achieved through the experimental design modifications suggested above are unlikely to be realized in chromosome arms carrying large inversions, whereby inversion-free populations may be necessary to ensure that such improvements are realized over the entire genome. Although inversion-free *D. melanogaster* populations can be obtained via karyotyping individuals prior to the study, the time and effort expended in such screens may become prohibitive if large founding populations are desired. Another possibility is to use an alternative *Drosophila* species for which segregating inversions are rare, such as *D. simulans* or *D. mauritiana*, instead of *D. melanogaster*. Although these species currently lack the detailed genetic and genomic resources available for *D. melanogaster*, the ongoing development of portable molecular tools has made functional validation of candidate loci in these species tenable (Carroll 2011; Liu et al. 2012; Yu et al. 2013).

## Conclusion

Previous *D. melanogaster* E&R studies have not directly addressed the role that inversions or long-range LD may have had in generating the large numbers of reported candidate SNPs. Our results imply that such estimates were probably inflated by false positives resulting from these phenomena. Consequently, *Drosophila* E&R study designs will need to be modified before the true causal loci that contribute to adaptation and/or complex traits can be reliably identified. While we have outlined several possibilities here, we also point out that the study by Kofler and Schlötterer (2013) is intended as a general guideline for E&R study design. There the authors use haplotype-based simulations to investigate E&R performance for wide range of experimental and evolutionary variables and thereby provide an invaluable reference for future *Drosophila* E&R research.

## Materials and Methods

### Experimental Populations and Evolution Regimes

Full details on the experimental setup are outlined in Orozco-terWengel et al. (2012). Briefly, wild *D. melanogaster* were collected in Northern Portugal in the summer of 2008. The population was maintained as 113 isofemale lines for five generations in the laboratory prior to the creation of mass-bred populations. Ten replicated mass-bred populations were created, with each replicate combining five females from each isofemale line. This initial population of merged isofemale lines is referred to as the base population or F0. Five replicate populations were maintained in each of two novel environments: one hot and one cold treatment. Each treatment cycled between two fixed temperature and light settings every 12 h—between 10 °C and 20 °C in the cold treatment and 18 °C and 28 °C in the hot treatment—with light and dark periods occurring with the hot and cold phase of each treatment, respectively. The heating/cooling period between the two fixed temperatures was relatively rapid, taking ∼1 h before the new temperature is reached, such that most time is spent at either of the modal temperatures. Each replicate was maintained at ∼1,000 individuals, with ∼50:50 sex ratios, that were evenly distributed across five ∼225 ml bottles. Each bottle contained ∼70 ml of standard *Drosophila* medium. Generations were nonoverlapping.

### Genome Sequencing and Mapping

Illumina full genome sequencing was performed on three replicates for each of the base population and evolved populations. For the hot treatment, a single replicate of the 23rd generation and two from the 15th generation were sequenced (Orozco-terWengel et al. 2012), whereas for the cold treatment all three sequenced replicates came from the 15th generation. Sequencing was performed using paired-end reads 75 bp in length in the hot treatment (GAIIx) and 100 bp long in the cold treatment (HiSeq). For each replicate and time point, ∼500 flies were pooled and sequenced as one sample (i.e., Pool-Seq; Futschik and Schlötterer 2010). Two additional replicates were also sequenced from the 15th generation of the hot and cold treatments. These were used to check the reproducibility of candidate detection as described below. Average genome-wide coverage ranged from ∼35- to ∼90-fold for each of the sequenced replicates (supplementary table S4, Supplementary Material online).

Mapping was carried out using a combination of purpose-built and third-party software, which is described in detail in Kofler, Orozco-terWengel et al (2011). Briefly, reads were trimmed to remove low-quality bases and then mapped with bwa (version 0.5.8c) (Li and Durbin 2009) against the *D. melanogaster* reference genome (version 5.18) and *Wolbachia* (NC_002978.6). The alignment files were converted to the SAM format (Li et al. 2009) with reads not mapped in proper pairs or with a mapping quality of less than 20 being removed. SAM files were converted into the pileup format and indels and repeat sequences identified by RepeatMasker 3.2.9 (www.repeatmasker.org, last accessed November 5, 2013) masked. The aligned pileup files were converted to a synchronized file (Kofler, Pandey et al. 2011) from which SNPs and their accompanying allele frequencies were derived. All allele frequency changes presented herein were averaged across all three replicates (weighted by coverage). We further removed all SNPs whose coverage was either among the top 2% of a given replicate and time point or had a minimum count less than 30 across the 23 populations in our extended data set that comprised additional replicates and time points that were not analyzed here. Because the average coverage per SNP across the extended data set was ∼1,500, the minimum count of 30 equates to an average 2% minor allele frequency across all replicates and time points (although allele frequencies could be lower in the time points that were specifically analyzed in this study). Annotation was based on version 5.40 of the *D. melanogaster* reference genome. Approximately 1.45 million SNPs were called overall.

### Effective Population Size, FDR, and Candidate Estimation

In order to estimate *N_e_* and FDR in both treatments, forward Wright–Fisher simulations were performed to generate expected neutral changes during the experiment. Three sets of neutral allele frequency changes were simulated for each treatment, matching the number of replicates in the study. For each simulation, 1.45 million SNPs were independently sampled (i.e., approximating the number of SNPs in our real data) from the distribution of allele frequencies expected after 15 generations of neutral evolution conditional on the starting frequency for each SNP. Starting frequencies for each SNP were randomly drawn from the empirical distribution of start frequencies in the experiment, with the frequencies being the coverage-weighted average across all replicates. The expected distribution of allele frequencies after 15 generations was created by generating an *N_i_* × *N_j_* transition matrix where each cell is the binomial probability of seeing *j* alleles with a starting frequency *i*/*N* (i.e., *N* is the effective population size) and then raising this matrix to the 15th power. To simulate the effects of DNA pooling in our study, an additional round of binomial sampling was conducted on the start and end allele frequencies for each SNP. To do this, each SNP was assigned a random coverage value that was drawn from the empirical distribution of coverages from a specific generation and treatment. This was done separately for each replicate, thereby capturing the heterogeneity in coverage both within and between replicates in the study.

The effective population size of the observed data was estimated as follows. First, multiple simulations were performed as outlined above, each with a differing *N_e_*. Each simulation produced a set of expected start and end allele frequencies for a given *N_e_*, from which an allele frequency change (AFC) distribution was obtained (i.e., the end frequency minus start frequency for all 1.45 million SNPs). The expected AFC distribution was then compared for goodness of fit with the observed AFC distribution by summing the squared difference of the AFC values evaluated in 1% frequency change bins. Because individual replicates were simulated, we averaged the goodness of fit across the three replicates to get a point estimate for each *N_e_*. Additionally, the error around this mean was sufficiently large for both treatments that the best-fitting *N_e_* was not significantly different from many neighboring *N_e_* values. Consequently, we took the lowest *N_e_* from amongst this range (∼250, supplementary table S1, Supplementary Material online) as it is expected to provide the most conservative estimate of the FDR (because the strength of drift increases as *N_e_* decreases). The *N_e_* for individual chromosomes was also inferred by limiting the observed data to that specific to each chromosome and then repeating the steps above.

Following *N_e_* estimation, the number of putative selected SNPs was inferred as follows. First, all ∼1.45 million SNPs that remained after filtering were subjected to the CMH test (Agresti 2002). The CMH test was shown to outperform several other statistics that were used for candidate inference in recent *Drosophila* E&R studies (Kofler and Schlötterer 2013). Testing was conducted separately for the hot and cold treatments, with each being contrasted against the base population. Second, the CMH test was applied to simulated SNP data that was derived using the conservative *N_e_* estimate of 250 (discussed earlier). Finally, the number of candidate SNPs was estimated by setting an empirical FDR of 0.001 for each treatment. Thus, the number of candidates for a given treatment was quantified as the number of observed SNPs that had CMH *P* values that were lower than the least significant CMH *P* value from among the 0.1% most extreme simulated SNPs. The number of candidate SNPs was also inferred for a range of different FDR values and the results are shown in supplementary table S1, Supplementary Material online.

To examine the effects that linkage between loci had on the candidate inference, we also performed 30 forward neutral simulations starting from a population with 250 unique haplotypes (i.e., 250 homozygous diploid individuals) for 15 generations. Thus, the number of founding haplotypes and the effective population size during the simulations was 250. Assuming that the isofemale founders maintained two segregating chromosomes per line on average—which is not unreasonable considering that they were five generations old when making the mass-bred populations—then these numbers roughly accord with those expected within our experimental populations. The simulations were performed using MimicrEE (Kofler and Schlötterer 2013). This software enabled the modeling of recombination at rates that were derived from empirical estimates (Fiston-Lavier et al. 2010). Thirty simulations were performed and divided into 10 equal-sized subsets. The three simulations from each of the 10 subsets were then downsampled to match the coverage distributions of the three empirical replicates from the relevant generation and treatment. Next, we applied the CMH test to all sites in each subset and estimated the number of candidates thereof via the FDR method outlined in the preceding paragraph. The haplotypes used in the simulations were created by neutral coalescent simulations and are described in Bastide et al. (2013).

### Enrichment in Thermal Tolerance Genes

We tested for treatment-specific evolution by looking for candidate enrichment in genes associated with heat and cold tolerance. Thermal tolerance gene sets were taken from the Candidate Stress Genes in *Drosophila* database, which was obtained from the Centre for Environmental Stress and Adaptation Research website (http://cesar.org.au/, last accessed November 5, 2013). Only genes with a significant and unique association with either heat or cold tolerance were included in the analyses (supplementary table S2, Supplementary Material online). Separate enrichment analyses were performed on the 2,000, 4,000, 6,000, 8,000, 10,000, 12,000, 14,000, 16,000, 18,000, 20,000, 25,000, 30,000, 35,000, 40,000, 45,000, 50,000, 60,000, 70,000, 80,000, 90,000, and 100,000 most significant SNPs in each treatment. Note that each successive set of SNPs was an aggregation of previous SNP sets and the subsequent set of most significant SNPs. For instance, the set that contained the top 4,000 SNPs comprised the set containing the top 2,000 SNPs along with the next 2,000 most significant SNPs. All enrichment analyses were performed using Gowinda software (Kofler and Schlötterer 2012). Gowinda generates *P* values by conducting multiple simulations in which the position of candidate SNPs are randomly chosen from the ∼1.45 million of SNPs scattered throughout the genome. The *P* value was then quantified as the proportion of simulations that had more genes specific to a given category (in our study, genes associated either with hot or cold thermotolerance) containing at least one candidate SNP than the observed data set (Kofler and Schlötterer 2012). In total, we performed 100,000 permutations for each set of candidate SNPs in each treatment and for each set of thermal tolerance genes. In each case, a gene containing multiple candidate SNPs was only counted once (i.e., the gene option of the mode argument). Gene annotations based on version 5.40 of *D. melanogaster* reference genome and only the full coding region of each gene and not the upstream or downstream flanking regions (i.e., the gene option of the gene-definition argument) were considered. Because the thermal genes were specifically tested for enrichment with the expectation that there would be enrichment for hot alleles in heat tolerance genes, and cold alleles in cold-tolerance genes, we report *P* values rather *q* values adjusted for testing multiple categories. This software was specifically designed to deal with differences in average gene size among categories and therefore does not result in the overrepresentation of categories with larger than average gene sizes (Kofler and Schlötterer 2012).

### Identification of Putative High LD Regions and Short Introns

LD can result in large number of false positives, for instance via allele frequency correlations between selected and neutral linked sites. Consequently, the following regions were designated as having putatively elevated LD and were removed from specific analyses as indicated in the main text. First, regions covered by inversions or within 500 kb either side of the inversion breakpoints. Segregating inversions were indentified in our experimental evolution populations by Kapun et al. (2013). Second, a 1 Mb region located within the inversion-free part of 3R. This region was found to harbor a large number of low-frequency alleles that showed a similar allele frequency change, a pattern consistent with selection upon a single haplotype block (Orozco-terWengel et al. 2012). Finally, regions with a local recombination rate less than 2 cM/Mb. The recombination rates were taken from Comeron et al. (2012). Approximately 300,000 of the original 1.45 million SNPs remained following the removal of these regions. Because 3R contained three segregating inversions and the putative selected haplotype block, only 15,000 SNPs remained following removal of these regions, whereas other chromosomes maintained at least 50,000 SNPs.

Short introns coordinates were taken from Lawrie et al. (2013). To further ensure that short introns sites were behaving neutrally, we only took those that were located within the putative low LD regions identified above.

### ROC Curves and Reproducibility

To test the reproducibility of our top candidates, two additional replicates were sequenced from generation 15 in each treatment, with all sites being processed through the same pipeline as the original candidates. Because we did not have additional replicates from the base population, two new base replicates were created. This was done by combining the reads from the separate base replicates into a single file, from which two thirds of all reads were randomly sampled (without replacement) and split in half to generate two new files each having one-third of the combined base population reads (∼50-fold coverage). All SNPs from the two new sets of replicates were then subjected to CMH testing and compared for consistency with the original data set using ROC curves. For the sake of consistency, the ∼1.45 million SNPs from the original data set were used in the comparisons.

We used a modified ROC curve analysis to provide a graphical representation of the consistency between the original and new data sets; that is, those based on three replicates and two replicates, respectively. The ROC curves quantify the overlap between the two data sets across successively larger groups of SNPs, with SNPs having been ranked by their CMH *P* value. Points falling on the 1:1 diagonal line indicate that overlap between the two sets of SNPs matches chance expectations of reproducibility, whereas points lying above the diagonal indicate excess reproducibility. Because we are interested in changes that were most likely to be caused by the deterministic process of selection as opposed to the stochastic effects of genetic drift, only SNPs where the same allele was increasing across all five replicates were considered as being reproducible.

The expected degree of reproducibility under neutrality was quantified using neutral MimicrEE simulations (Kofler and Schlötterer 2013). All parameters used were identical to the simulations used for the FDR estimates outlined above, except that a total of 50 data sets were simulated in this case. The simulated data sets were divided into 10 equally sized subsets, with each subset being further split into a group of three replicates and a group of two replicates. Following downsampling of the data sets to match our empirical coverage distributions (discussed earlier), for each set of simulations CMH tests were applied to each group of three and two replicates. The resulting data were compared for reproducibility using ROC curves as above.

### LD Analyses

The degree of LD around the top 2,000 candidates was estimated for both treatments following the same methods outlined in Orozco-terWengel et al. (2012). Briefly, we estimated the median *P* value for all SNPs flanking the top 2,000 candidates in nonoverlapping 50-bp windows either side of the focal candidates. The decay in the *P* value around the focal candidates provides a proxy for the strength and extent of LD in regions flanking putative selected sites. To ascertain the background level of LD among similar SNPs, 2,000 more SNPs were randomly drawn from the genome after excluding all regions within 200 bp of the top 2,000 candidates. The numbers of random SNPs drawn from each chromosome arm matched the distribution of the top 2,000 candidates in each treatment.

We used MimicrEE simulations (Kofler and Schlötterer 2013) to estimate the degree of long-range LD achievable given a simple scenario where strong selection acted on a singleton located in a single haplotype. The selection strength (0.5) was based on increasing a single allele from 1% to 30% over 15 generations (i.e., the mean allele frequency change observed for the top 2,000 candidate SNPs in the hot treatment). This was derived using *s* = 2ln(*p*_0_*q*_1_/*p*_1_*q*_0_)/Δ*t*, where *s* is the selection coefficient, *p* and *q* the biallelic frequencies at the start (subscript 0) and end (subscript 1) generations, and Δ*t* is the change in generations (eq. 3.3 in Charlesworth and Charlesworth [2010]). Other than the selected site, all other parameters were the same as the neutral simulations outlined above. Nine simulations, in which the singleton increased in frequency by 25–35%, were included in the analyses. These were evenly split into three subsets, with each subset being downsampled and submitted to CMH testing (in the same manner as the neutral simulations discussed earlier). The resulting data were then used to produce Manhattan plots that enabled visual assessment the degree of long-range LD (fig. 7).

## Supplementary Material

Supplementary figures S1–S4 and tables S1–S4 are available at *Molecular Biology and Evolution* online (http://www.mbe.oxfordjournals.org/).

Supplementary Data
