# Supplementary material for: Massive Habitat-Specific Genomic Response in D. melanogaster Populations during Experimental Evolution in Hot and Cold Environments
Source: Mol Biol Evol. 2013 Oct 22;31(2):364–75. doi: 10.1093/molbev/mst205 (PMC3907058; doi:10.1093/molbev/mst205)
Supplement: Supplementary Data [file supp_31_2_364__index.html]

Massive habitat-specific genomic response in D. melanogaster populations during experimental evolution in hot and cold environments — Massive Habitat-Specific Genomic Response in D. melanogaster Populations during Experimental Evolution in Hot and Cold Environments — Massive Habitat-Specific Genomic Response in D. melanogaster Populations during Experimental Evolution in Hot and Cold Environments — Supplementary Data 

# Massive Habitat-Specific Genomic Response in *D. melanogaster* Populations during Experimental Evolution in Hot and Cold Environments

## Supplementary Data

files

**Files in this Data Supplement:**

- Supplementary Data - doc file
