# Supplementary material for: Massive Habitat-Specific Genomic Response in D. melanogaster Populations during Experimental Evolution in Hot and Cold Environments
Source: Mol Biol Evol. 2013 Oct 22;31(2):364–75. doi: 10.1093/molbev/mst205 (PMC3907058; doi:10.1093/molbev/mst205)
Supplement: Supplementary Data [file supp_mst205_F15HotCold_comparison_Supplement.doc]

**Supplementary tables and figures**

**Table S1.** Estimated number of candidates for a given false discovery rate and effective population size (*N_e_*). Estimates based on simulations of neutral evolution where each SNP evolved independently (Independent SNPs) or SNPs were linked on haplotypes (Haplotypes). Hapolotype based simulations results are shown for all ~1.45 million SNPs (All) or with low recombining regions removed (<2cM/Mb; High RR). The haplotype simulation values are the average over the ten independent sets of simulations. Entries in bold refer to number of candidates based on the best set of *N_e_* estimates (see Methods). The difference in the number of inferred candidates between the two simulation types probably arises from the independent SNPs involving millions of simulations on a SNP by SNP basis, whereas only 10 sets simulations that were performed for the haplotype data. This meant that extreme allele frequency changes were much more common for the individual simulations.

| Simulations | Treatment | *N_e_* | Region | FDR.0.001 | FDR.0.005 | FDR.0.01 | FDR.0.02 | FDR.0.05 | FDR.0.1 |
| --- | --- | --- | --- | --- | --- | --- | --- | --- | --- |
| Independent SNPs | Hot | 200 | – | 10824 | 30140 | 46793 | 74066 | 138433 | 226499 |
|  |  | 225 | – | 13734 | 35306 | 53961 | 83654 | 153526 | 245369 |
|  |  | **250** | – | **17085** | **41684** | **61899** | **94983** | **168709** | **264298** |
|  |  | **275** | – | **19410** | **46954** | **69611** | **104639** | **181577** | **280101** |
|  |  | **300** | – | **22571** | **52530** | **76718** | **113447** | **192479** | **293454** |
|  |  | **325** | – | **26189** | **57532** | **83133** | **121403** | **203618** | **306771** |
|  |  | **350** | – | **28475** | **62975** | **90023** | **129454** | **213828** | **319075** |
|  |  | 375 | – | 31164 | 67595 | 96146 | 137548 | 224326 | 330396 |
|  |  | 400 | – | 33735 | 71727 | 101002 | 143477 | 231954 | 340069 |
|  | Cold | 200 | – | 1589 | 7907 | 15805 | 31462 | 76282 | 150864 |
|  |  | 225 | – | 2452 | 10967 | 20775 | 39002 | 90452 | 171700 |
|  |  | **250** | – | **3594** | **14129** | **26176** | **47092** | **103644** | **189419** |
|  |  | **275** | – | **4752** | **18018** | **31396** | **54959** | **117212** | **207164** |
|  |  | **300** | – | **5858** | **21410** | **36640** | **62816** | **128831** | **223433** |
|  |  | **325** | – | **7009** | **24276** | **41017** | **69432** | **139662** | **236602** |
|  |  | **350** | – | **8321** | **27911** | **46414** | **76905** | **150763** | **250621** |
|  |  | 375 | – | 10291 | 31897 | 51647 | 83975 | 160148 | 262699 |
|  |  | 400 | – | 11749 | 35228 | 56201 | 90234 | 169618 | 273816 |
| Haplotypes | Hot | 250 | All | 39064.3 | 81910.9 | 114437.7 | 161002 | 255962.9 | 370345.8 |
|  | Cold | 250 | All | 23439.2 | 60813.6 | 92046.4 | 139120.1 | 237840.6 | 356922.8 |
|  | Hot | 250 | High RR | 39062.2 | 81982.1 | 114507.2 | 161080.8 | 256053.4 | 370398.5 |
|  | Cold | 250 | High RR | 23449.2 | 60887.4 | 92120.3 | 139220.4 | 237937.8 | 356980.9 |

**Table S2.** List of categorized thermal tolerance genes used in the present study. All genes were taken from CESAR website ([www.cesar.org.au](http://cesar.org.au/index.php?option=com_candidate_gene)); an online dataset of genes associated with stress response in *D. melanogaster* that are derived from published studies. The categories indicate whether the gene was uniquely associated with heat or cold tolerance (genes common to the two thermal stress types were removed). Names refer to the Flybase ID for each gene. Genes in bold are those containing hot (cold) candidate SNPs from most significantly enriched heat (cold) tolerance category (i.e. the top 8000 hot candidates and top 6000 cold candidates).

| Category | Gene symbol |
| --- | --- |
| Heat tolerance | CG7435 CG5683 CG3481 **CG1462** CG1072 **CG6605** CG1567 CG10026 CG10189 CG10383 CG10472 CG10477 CG10513 CG10514 CG10592 CG10680 **CG10825** CG11034 CG11200 CG11314 CG11315 CG11378 CG11444 **CG43462** CG11722 CG12116 CG12288 CG12374 CG12656 CG12813 CG13083 CG13084 **CG13086** CG13095 CG13252 CG13309 CG13322 CG13450 CG13492 CG13607 CG13833 CG14120 CG14439 CG14527 CG14528 CG14629 CG14804 CG14872 CG14906 CG14935 CG15065 CG15141 CG15201 CG15247 CG15737 CG1583 CG16743 CG16749 CG16762 CG16799 CG16898 CG16985 CG16986 CG17108 **CG17124** CG17224 **CG17475** CG1753 CG17633 CG17724 **CG17751** CG17752 **CG17836** CG17931 CG1809 CG18249 CG18327 CG18493 **CG18522** CG18585 CG1946 CG2065 CG2186 CG2254 CG2694 CG2736 CG2811 CG30035 CG30359 **CG30502** CG3106 CG31104 CG31148 CG3246 CG32687 CG3270 CG32736 CG10096 CG10097 **CG3301** CG3332 **CG3344** CG3348 **CG3409** **CG3734** CG3739 **CG3940** CG3984 CG4266 CG4301 CG4847 CG4877 CG4989 **CG5023** CG5053 CG5107 CG5150 CG42329 CG5162 **CG5205** CG5384 CG5390 CG5618 CG5791 CG5804 CG5945 **CG5966** CG5969 CG6012 CG6126 **CG6164** CG6271 CG6295 **CG6426** CG6484 CG6483 CG6579 CG6602 CG6660 **CG6726** **CG6733** CG7025 CG7130 CG7203 CG7300 CG7443 **CG7601** CG7616 CG7882 CG7891 CG7916 CG7953 CG8093 CG8147 **CG8176** CG8235 CG8249 CG8289 CG8317 CG8520 CG8560 CG8562 CG8693 CG8773 **CG8774** CG8834 CG8891 CG8997 CG9259 CG9394 CG9466 CG9468 CG9497 CG9646 CG9673 CG9682 CG9928 CG1049 CG13664 CG8610 CG12019 **CG6384** CG7281 **CG3360** CG17970 CG6730 CG2397 CG13977 CG10240 CG17903 CG16792 CG3210 CG12363 CG11901 CG6341 CG8365 CG6917 CG4205 CG8261 CG9280 CG4181 CG12242 CG5164 CG17522 CG17523 CG17524 CG17533 CG4167 CG4460 CG4466 CG5436 CG18743 CG4147 CG5834 CG4472 **CG3953** CG10369 CG4715 CG6298 CG31034 CG15106 CG8696 CG8695 CG6955 CG17342 CG17397 CG9470 CG4082 CG4123 CG7438 **CG9155** CG5258 CG12092 CG32190 CG5994 CG4007 CG8782 CG5581 CG4799 CG17725 CG8251 CG4033 CG34374 CG8418 CG3178 **CG9668** CG6253 CG3203 CG4863 CG4464 CG6779 CG2381 CG5723 CG7052 CG8846 CG1841 CG30404 CG3171 CG31509 CG5609 CG31508 CG33117 CG31691 CG14027 CG31193 CG6720 CG6492 **CG4548** **CG6875** CG31628 CG3705 **CG10422** CG3897 **CG1759** CG5363 CG5582 CG1772 CG9908 CG1725 **CG6667** CG1828 CG3727 CG2096 **CG31000** CG1594 CG7754 CG33956 CG10072 CG12350 CG3018 CG32597 **CG7832** CG15002 CG6936 CG7494 CG10406 CG2302 CG6824 CG8705 CG32885 **CG7904** CG3027 CG9412 CG10360 CG7642 CG9533 CG1891 CG7113 CG6781 **CG3992** **CG7951** CG9131 CG9675 CG32130 CG3850 CG2194 CG31057 CG7855 CG10034 CG16724 CG2155 |
| Cold tolerance | CG10912 CG13510 CG15347 CG15745 CG42318 CG18609 CG2118 **CG33970** CG3124 CG32038 **CG3345** **CG3814** CG5011 CG8012 CG8838 **CG9568** CG3132 CG4193 **CG9885** CG2086 CG9355 CG4143 **CG5403** CG10203 |

**Table S3.** Genomic distribution of all ~1.45M SNPs and the top 2000 candidates in both treatments. Red and blue text indicates chromosome arms that were enriched for candidates in the hot and cold treatment, respectively.

|  |  | Genome-wide | | Hot candidates | | Cold candidates | |
| --- | --- | --- | --- | --- | --- | --- | --- |
| Dataset | Region | Count | Proportion | Count | Proportion | Count | Proportion |
| All SNPs | X | 161208 | 0.111 | 7 | 0.004 | 68 | 0.034 |
|  | 2L | 363788 | 0.250 | 202 | 0.101 | 617 | 0.309 |
|  | 2R | 249126 | 0.171 | 94 | 0.047 | 390 | 0.195 |
|  | 3L | 327373 | 0.225 | 129 | 0.065 | 100 | 0.050 |
|  | 3R | 350692 | 0.241 | 1561 | 0.781 | 825 | 0.413 |
|  | Other | 4934 | 0.003 | 7 | 0.004 | 0 | 0.000 |

**Table S4.** Coverage for all major chromosomal regions and all sequenced populations used in the main analyses. All replicates for the hot and cold treatment were sequenced in generation 15, except replicate 1 for the hot treatment which was sequenced at generation 23. For all populations, replicates 1, 2 and 3 were used for the main analyses, while replicates 4 and 5 were used to analyze repeatability (two new base populations were created by randomly drawing reads from the combined base population data; see Methods).

|  |  | Genomic region | | | | | |
| --- | --- | --- | --- | --- | --- | --- | --- |
| Popualtion | Replicate | 2L | 2R | 3L | 3R | X | All |
| Base | 1 | 76.51 | 75.88 | 75.90 | 75.58 | 73.68 | 75.72 |
|  | 2 | 35.09 | 34.80 | 34.94 | 34.63 | 33.89 | 34.76 |
|  | 3 | 59.89 | 61.80 | 59.14 | 61.39 | 57.47 | 60.11 |
| Hot | 1 | 45.02 | 45.60 | 44.46 | 45.18 | 43.12 | 44.80 |
|  | 2 | 58.54 | 57.97 | 58.10 | 57.88 | 56.91 | 58.01 |
|  | 3 | 50.56 | 50.94 | 50.01 | 50.71 | 48.96 | 50.34 |
|  | 4 | 44.38 | 44.98 | 44.76 | 44.77 | 44.09 | 44.62 |
|  | 5 | 34.93 | 35.14 | 35.09 | 34.99 | 34.72 | 34.99 |
| Cold | 1 | 80.80 | 81.41 | 80.32 | 80.53 | 78.13 | 80.42 |
|  | 2 | 90.58 | 90.59 | 90.14 | 90.07 | 87.63 | 90.02 |
|  | 3 | 63.74 | 63.93 | 63.63 | 63.61 | 62.29 | 63.54 |
|  | 4 | 63.99 | 64.52 | 64.33 | 64.27 | 63.33 | 64.14 |
|  | 5 | 49.78 | 50.16 | 49.98 | 49.97 | 49.29 | 49.88 |


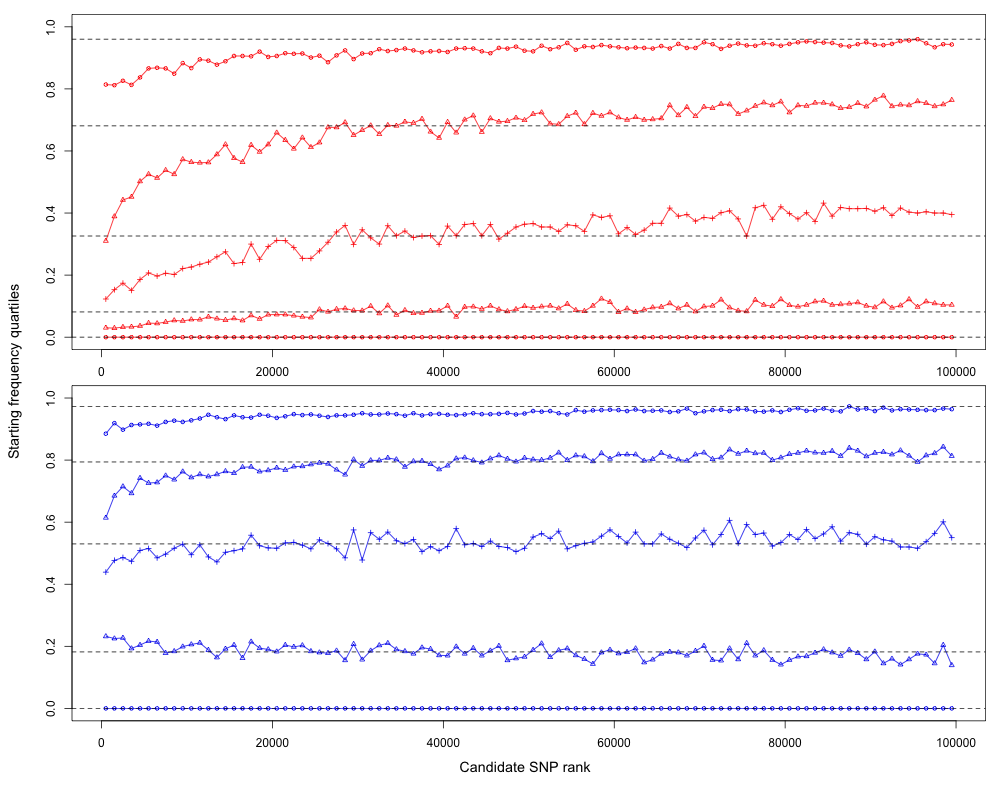


**Figure S1.** Starting allele frequency quartiles (i.e. minimum, 25%, 50%, 75% and maximum values) for both, the hot and cold treatments across successive sets of 1000 SNPs ranked by *p*-value. Hence, the first set of points denotes the top 2000 candidates, the next set the next best 2000 and so on, for the top 100K SNPs. Starting allele frequencies are skewed towards low values in the hot treatment, and toward intermediates values in the cold treatment for the top ~30,000 ‘candidate’ SNPs. Dashed lines are genome-wide averages for each quartile.


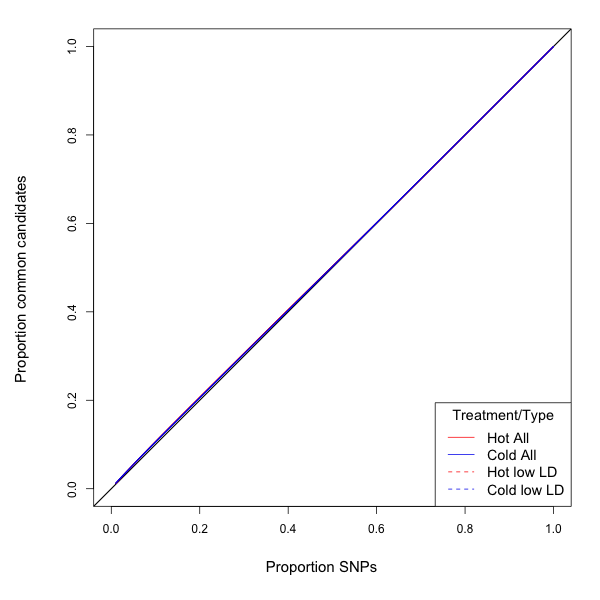


**Figure S2.** ROC curve indicating the proportion of common candidate SNPs shared between two independent sets of derived populations relative to cumulatively larger sets of ranked SNPs for simulated data (see Methods). Cumulative sets of SNPs, ranked by *p*-value are plotted on the x-axis, the proportion of common SNPs on the y-axis. The diagonal black line indicates expected values of overlap, whereby simulated data do not deviate from expectations. Note that there was no observable difference between datasets that included all SNPs and those from which SNPs in regions of putative low LD were excluded (see Methods).


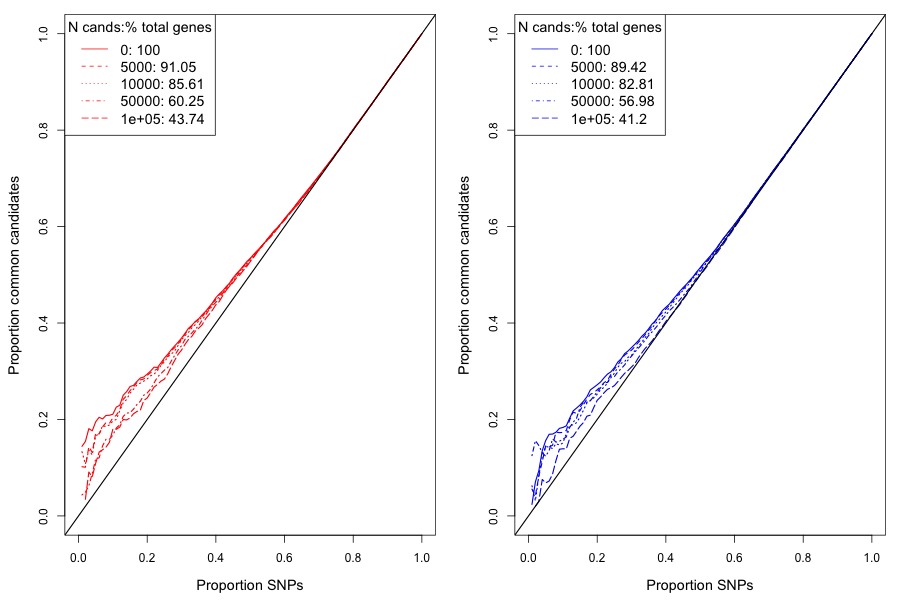


**Figure S3.** As for Figure S2, with the exception that only short introns are included (see Methods). Because the black line indicates expected values of overlap and short introns are expected to act in a neutral fashion in *D. melanogaster*, there are more common SNPs in short introns than expected. This excess is still present after removing short introns from genes having a significant candidate SNP. The number of candidate SNPs considered and the percentage of total genes remaining after removal of genes containing these SNPs is indicated in the key.


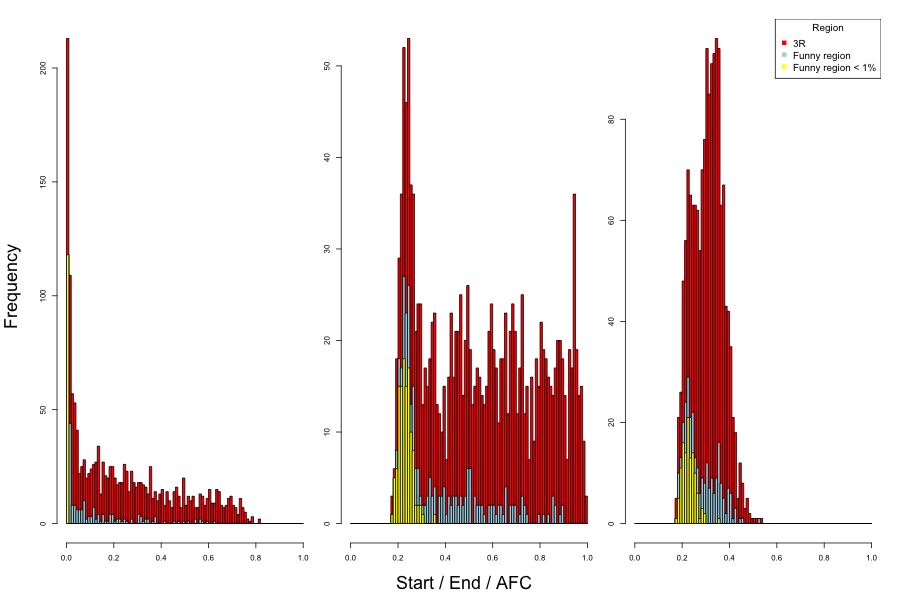


**Figure S4.** The start (left panel) and end (middle panel) distribution of allele frequencies for the top 2000 hot candidates that are located in chromosome arm 3R (red), within the rare putative haplotype block in this chromosome arm (blue), and with a starting frequency less than 1% in the rare haplotype (yellow). There is a notable lack of variation in allele frequency change amongst sites that start at a frequency less than 1% (right panel), a signal that is synonymous with selection on a rare haplotype.
